# Supplementary material for: Service reliability evaluation of highway tunnel based on digital image processing
Source: PLoS One. 2023 Aug 11;18(8):e0288633. doi: 10.1371/journal.pone.0288633 (PMC10420349; doi:10.1371/journal.pone.0288633)
Supplement: Attachment — Submitted filename: Response to Reviewers.docx [file pone.0288633.s002.docx]

Firstly, we would like to thank you for your kind letter and for reviewers’ constructive comments concerning our article (Manuscript No.: PONE-D-22-32743).These comments are all valuable and helpful for improving our article. All the authors have seriously discussed about all these comments. According to the reviewers’ comments, we have tried best to modify our manuscript to meet with the requirements of your journal. In this revised version, changes to our manuscript within the document were all highlighted by using red colored text. Point-by-point responses to the reviewers are listed below this letter.

1. There are two same grades in 5 grades of tunnels’ safety grades in abstract.

In view of this problem, the safety grades mentioned in the whole paper are revised and uniformly defined as normal, degraded, inferior, deteriorated and hazardous.

2. 1.3 section has appeared for twice in your article, please check.

In response to this issue, the section numbering in the article has been revised.

3. Parameters in 1.3 should be defined, such as .

The parameters $x_{i}$、$y_{i}$, which were not mentioned in section 1.3, have been defined. $(x_{i},y_{i})$ is the intersection point between the crack and the left line of the fine block, $(x_{i+1},y_{i+1})$ is the intersection point between the crack and the right line of block.

4. In Section III, level 3 is same as level 4.

In view of this problem, the safety grades mentioned in the whole paper are revised and uniformly defined as normal, degraded, inferior, deteriorated and hazardous.

5. Before conducting the EM clustering, you had better to check your features’ range. Especially the distance-based algorithms, the feather with high range may have bigger influence on your results. Considering to standardize or normalize each features in Section III.

This problem has not been modified because the disease data of tunnel samples is real data with a large variation between samples. If standardized or normalized processing is performed, the safety classification will be inaccurate due to the reduced variation between samples.

6. The disease description in your table 3, normal level should be in normal service instead of not in normal service.

There is a writing error here. The correct sentence should be "The number of cracks is relatively small, indicating normal service."

7. It makes me confused that you conduct the PLSR to evaluate the tunnels’ safety after using EM clustering to obtain appropriate the levels of disease and using Euclidean distance range to explain it. Is It simpler to use your evaluation method than the computation of Euclidean distance? In your section 3, you can obtain the actual safety level of the tunnel by calculating the Euclidean distance of your variances. The Section 4 seems like no contribution to the others. Why is your Section 4 necessary for this article?

The third section categorizes the safety level of tunnels using clustering methods, but it does not provide a description of the degree of damage of the samples. Instead, the degree of damage is differentiated by calculating the Euclidean distance between samples. In the fourth section, a reliability evaluation formula is proposed for tunnel linings, based on the determined classification levels and the corresponding degree of damage.

The issue has been corrected in the original text, with an additional paragraph added to the fourth section to provide an explanation.

8. Your variances are the fracture length, maximum width and fractal dimension respectively before the Section 4, and variances become to cumulative value in Section 4. Please be consistent if you make mistakes of expression.

This article uses cumulative values, and the crack length, maximum width, and fractal dimension in Table 1 are all cumulative values of tunnel defect samples. To avoid any misunderstanding, the original Table 1 has been modified, with the crack length and maximum width changed to "cumulative crack length" and "cumulative crack width," respectively.

9. HSR is proposed as , so the HSR may be . Substituting the sample number 1’s features ( ) into HSR, I get whereas the HSR calculated result is 375.47 in table 4. It also makes me confused. Please explain it to me. The same questions also appear in table 8.

The partial least squares regression equation constructed in this article is $HSR'=\alpha L+\beta W+\lambda FW+C$. The constant term C has a numerical value of 375.456, which has been rounded to 375.47 in the table to maintain consistency in the format.
